# Surveillance for Azole-Resistant *Aspergillus fumigatus* in a Centralized Diagnostic Mycology Service, London, United Kingdom, 1998–2017

**DOI:** 10.3389/fmicb.2018.02234

**Published:** 2018-09-20

**Authors:** Alireza Abdolrasouli, Michael A. Petrou, Hyun Park, Johanna L. Rhodes, Timothy M. Rawson, Luke S. P. Moore, Hugo Donaldson, Alison H. Holmes, Matthew C. Fisher, Darius Armstrong-James

**Affiliations:** ^1^Diagnostic Mycology Service, Department of Medical Microbiology, North West London Pathology, Imperial College Healthcare National Health Service Trust, London, United Kingdom; ^2^Fungal Pathogens Laboratory, National Heart and Lung Institute, Imperial College London, London, United Kingdom; ^3^Department of Medicine, Imperial College London, London, United Kingdom; ^4^MRC Centre for Global Infectious Disease Analysis, Imperial College London, London, United Kingdom; ^5^National Institute for Health Research Health Protection Research Unit in Healthcare Associated Infections and Antimicrobial Resistance, Imperial College London, London, United Kingdom; ^6^Imperial College Healthcare National Health Service Trust, London, United Kingdom; ^7^Chelsea and Westminster National Health Service Foundation Trust, London, United Kingdom

**Keywords:** *Aspergillus fumigatus*, azole resistance, *cyp51A*, antifungal agents, surveillance

## Abstract

**Background/Objectives:** *Aspergillus fumigatus* is the leading cause of invasive aspergillosis. Treatment is hindered by the emergence of resistance to triazole antimycotic agents. Here, we present the prevalence of triazole resistance among clinical isolates at a major centralized medical mycology laboratory in London, United Kingdom, in the period 1998–2017.

**Methods:** A large number (*n* = 1469) of clinical *A. fumigatus* isolates from unselected clinical specimens were identified and their susceptibility against three triazoles, amphotericin B and three echinocandin agents was carried out. All isolates were identified phenotypically and antifungal susceptibility testing was carried out by using a standard broth microdilution method.

**Results:** Retrospective surveillance (1998–2011) shows 5/1151 (0.43%) isolates were resistant to at least one of the clinically used triazole antifungal agents. Prospective surveillance (2015–2017) shows 7/356 (2.2%) isolates were resistant to at least one triazole antifungals demonstrating an increase in incidence of triazole-resistant *A. fumigatus* in our laboratory. Among five isolates collected from 2015 to 2017 and available for molecular testing, three harbored TR_34_/L98H alteration in the *cyp51A* gene that are associated with the acquisition of resistance in the non-patient environment.

**Conclusion:** These data show that historically low prevalence of azole resistance may be increasing, warranting further surveillance of susceptible patients.

## Introduction

*Aspergillus fumigatus* is a ubiquitous ascomycete mold and the primary etiologic agent of aspergillosis which varies in severity and clinical presentation. These manifestations include a spectrum of conditions including colonization, allergic response in allergic bronchopulmonary aspergillosis, chronic pulmonary aspergillosis, aspergilloma, and to the most severe form, invasive aspergillosis (Kosmidis and Denning, 2015). Sensitization to *Aspergillus* in patients with severe asthma is another form of disease. *Aspergillus* bronchitis is a recently described condition, especially in patients with cystic fibrosis (CF) or bronchiectasis, lung transplant recipients, and those receiving mechanical ventilation in intensive care units (Kosmidis and Denning, 2015). Moreover, aspergillosis may occur in immunocompetent hosts with influenza infection (Crum-Cianflone, 2016).

Triazoles have been the most widely used antifungal agents in prophylaxis and treatment of *Aspergillus*-related infections (Verweij et al., 2015). The Infectious Diseases Society of America (IDSA) guideline recommends the triazole antifungal voriconazole as the first line agent for the primary treatment of IA (Patterson et al., 2016). Since the late 2000s there has been a steady increase in the number of reported resistance to azole antifungals in *A. fumigatus*, causing a major clinical concern with subsequent treatment failure among some patients (Verweij et al., 2007; Chowdhary et al., 2013). The emergence and global spread of azole-resistant isolates led to a fundamental question as to whether first line clinical use of mold-active triazoles can be retained (Verweij et al., 2015).

The molecular basis of resistance to triazoles in *A. fumigatus* mainly involves the environmentally driven polymorphism TR_34_/L98H, which consists of a tandem repeat (TR) of 34 bases in the promoter of the *cyp51A* gene and a leucine-to-histidine change at codon 98 (Mellado et al., 2007); this polymorphism is globally widespread in environmental and clinical isolates (Chowdhary et al., 2015, 2017). Another *cyp51A*-mediated resistance alteration that leads to high-level voriconazole resistance, TR_46_/Y121F/T289A, has also been described in *A. fumigatus* (van der Linden et al., 2013). In contrast, non-synonymous mutations in the *cyp51A* gene cause structural alterations due to amino acid substitutions and are sufficient to induce resistance to some or all triazole drugs. Numerous mutations in *cyp51A* have been reported that confer resistance to triazoles *in vitro*. These resistant mutations often evolve during prolonged azole treatment in patients with chronic forms of aspergillosis (Hagiwara et al., 2016). In *A. fumigatus*, *cyp51A* gene encodes lanosterol 14-α-sterol demethylase which is required for the biosynthesis of ergosterol, an essential component of the fungal cell membrane (Chowdhary et al., 2014).

The true prevalence of azole resistance in *A. fumigatus* is largely unknown and multiple factors including sample size, method of detection and geographical differences in the studied samples might affect the prevalence rate of azole-resistant isolates (Verweij et al., 2016a). Overall azole resistance rates of 0–27.8% have been determined from different surveys (Vermeulen et al., 2013; Hagiwara et al., 2016; Chowdhary et al., 2017). Despite the global emergence of triazole resistance, the prevalence data on azole-resistant *A. fumigatus* in the United Kingdom is limited to reports (Howard et al., 2009; Bueid et al., 2010; Denning et al., 2011) generated by the National Aspergillosis Centre in Manchester. Howard and co-workers (Howard et al., 2009) have reported an increase in azole resistance (5%) in clinical *A. fumigatus* isolates since 2004. Later, another alarming increase in azole resistance frequency to 14% in 2008 and 20% in 2009 was reported by Bueid et al. (2010). Recent published data from the Public Health England, showed 8, 7, and 4.5% of clinical isolates referred to the National Mycology Reference Laboratory in, 2016 were resistant to itraconazole, posaconazole, and voriconazole, respectively (Public Health England, 2017), though the mechanism of resistance among these azole-resistant isolates remained unknown.

We hypothesized that prevalence of azole-resistant *A. fumigatus* reported by specialist or referral centers may not represent the true prevalence of azole resistance occurring in other institutions with different patient populations, thus surveillance studies at regional level are warranted. Our centralized mycology laboratory provides diagnostic service to eight major hospitals with mixed specialties in North West London, plus over 100 primary care providers, all serving a population of 2.5 million. The main patient population at risk of invasive fungal infections is diverse with high adult and pediatric hematology, oncology, renal transplant and intensive care caseloads. To investigate the prevalence of azole-resistance in clinical *A. fumigatus* isolates, antifungal susceptibility profiles of a large collection of unique clinical isolates of *A. fumigatus* collected over 17 years were retrospectively reviewed.

## Materials and Methods

### Data Collection

Our objective was to investigate the prevalence of azole-resistance in *A. fumigatus* isolates in a major centralized diagnostic mycology service based at the Imperial College Healthcare National Health Service trust, which provides diagnostic mycology service to the North West London area. Retrospective data on antifungal susceptibility profiles of a large collection of clinical *A. fumigatus* isolates tested between January 1998 – December 2017 was extracted from the laboratory database. This database is populated with antifungal minimum inhibitory concentration (MIC) data against fungal isolates cultured from diverse clinical samples from a mixed and unselected patient population. No data were available for period of January 2012– December 2014 as no susceptibility testing was carried out for molds during this period.

### Fungal Isolates

All isolates were identified as *A. fumigatus* species complex based on culture colonial morphology and microscopic characteristics. Adhesive tape technique was used for microscopic examination of fungal cultures. In addition, growth at 45°C was used to exclude most cryptic species. The identification of isolates with elevated azole MICs was confirmed by matrix-assisted laser desorption ionization–time of flight mass spectrometry (MALDI–TOF MS). Identification by MALDI–TOF MS was performed with a Microflex LT system (Bruker Daltonics, Bremen, Germany) using Biotyper 3.0 software with the additional fungi library (Bruker Daltonics, Bremen, Germany) according to the manufacturer’s recommendations. Exact identification of azole-resistant *A. fumigatus* isolates was confirmed by sequencing the partial calmodulin gene (*CaM* locus) as previously described (Samson et al., 2014).

### Antifungal Susceptibility Testing (AFST)

Antifungal susceptibility testing was carried out according to the CLSI M38-A2 standard broth microdilution method (Clinical and Laboratory Standards Institute, 2008) for filamentous fungi (isolates tested 1998–2011) and EUCAST EDEF 9.1 (Arendrup et al., 2012) (isolates tested 2015–2017). MICs were read at 48 h as the concentration of drug that elicited 100% inhibition of growth (amphotericin B, itraconazole, posaconazole, voriconazole) or as the minimum effective concentration (MEC) for caspofungin, anidulafungin, micafungin, in which the end-point is read as the lowest concentration at which the fungal hyphae can be seen to be stunted with swollen tips using an inverted microscope. For interpretation of MIC values, the EUCAST clinical breakpoints for *A. fumigatus* were used (Arendrup et al., 2012). For itraconazole, voriconazole, and amphotericin B MICs of ≤1 mg/L (susceptible) and >2 mg/L (resistant) and posaconazole MICs of ≤0.125 mg/L (susceptible) and >0.25 mg/L (resistant). No clinical breakpoints for the echinocandins have yet been established for *Aspergillus*. Quality control for AFST was ensured by testing the following type strains: *Candida parapsilosis* ATCC 22019, *Candida krusei* ATCC 6258, *A. fumigatus* NCPF 7097 and *A. fumigatus* NCPF 7100.

### Fungal DNA Extraction

Fungal genomic DNA was extracted as previously described (Abdolrasouli et al., 2015). Briefly, gDNA was extracted with an optimized MasterPure yeast DNA purification kit (Epicentre Biotechnologies, Cambridge, United Kingdom) with an additional bead-beating step included. Harvested conidia were homogenized using 1.0-mm-diameter zirconia/silica beads (BioSpec Products, Bartlesville, OK, United States) in a FastPrep-24 system (MP Biomedicals, Solon, OH, United States) at 4.5 m/s for 45 s. DNA samples were stored at −80°C for molecular testing.

### Genotype Testing

Five isolates collected from 2015 to 2017 with raised MICs to at least one triazole agent were available for molecular analysis. A commercially available real-time PCR and high-resolution melt-curve analysis, AsperGenius^®^ (PathoNostics, Maastricht, Netherlands) was utilized to detect alterations in *cyp51A* conferring resistance to triazole antifungal agents. The AsperGenius^®^ resistance multiplex assay targets the single-copy *cyp51A* gene of *A. fumigatus* and detects the TR_34_, L98H, Y121F, and T289A mutations to differentiate wild-type from mutant *A. fumigatus* isolates *via* melting curve analysis. This real-time PCR was performed according to the manufacturer’s instructions. The detection of four different fluorescent labels (emission spectra, 495 nm, 530 nm, 598 nm, and 645 nm) was enabled by using the Rotor-Gene Q (Qiagen, Heidelberg, Germany) for all experiments.

### Statistical Analysis

Categorical variables were reported as counts and percentages and were compared using Fisher’s exact tests. Statistical analyses were two sided, and *P* < 0.05 was considered to have statistical significance. Analyses were performed using GraphPad Prism software (version 6.0; GraphPad Software, La Jolla, CA, United States).

## Results

### Fungal Isolates

A total of 1,469 fungal isolates identified as *A. fumigatus* at the diagnostic mycology service based in North West London between 1998 and 2017 included 12 isolates with (minimum inhibitory concentrations) MICs above the breakpoints for itraconazole, posaconazole and/or voriconazole. Due to difference in antifungal susceptibility testing methodology (methods), results were presented in two time periods (retrospective 1998–2011; prospective 2015–2017).

### Retrospective Surveillance

From 1998 to 2011, a total of 1,151 isolates were identified as *A. fumigatus*. Respiratory samples were the most common (966/1151, 84%) source of isolation. Overall 0.43% (5/1151) of *A. fumigatus* isolates from five patients displayed elevated MICs to triazole antifungal agents. **Table 1** summarizes the characteristics and MIC results of resistant isolates in this study. For itraconazole, 3/1151 isolates had MIC values above the sensitive breakpoint (MIC > 2 mg/L). For voriconazole, 3/1151 were classified as resistant (MIC > 2 mg/L). Among 720 isolates tested against posaconazole, three isolates displayed reduced susceptibility (MIC ≥ 0.25 mg/L). No isolate in this collection displayed high level of resistance (MIC > 16 mg/L) to three tested triazole antifungal agents.

**Table 1:** Characteristics of *A. fumigatus* isolates of this study.

Case	Year	Gender/Age/Underlying disease	Sample type	Azole therapy	MIC or MEC (mg/L)					AFST method	*cyp51A* mutation
					ITC	VRC	PCZ	AMB	CAS		
1	2001	Gender/age unknown HIV	Sputum	Unknown	0.12	4	ND	0.25	ND	CLSI M38-A2	ND
2	2007	M/63, respiratory	Sputum	Unknown	4	0.5	0.25	0.06	0.007	CLSI M38-A2	ND
3	2008	M/34, hematology	Sputum	Unknown	8	4	2	0.25	8	CLSI M38-A2	ND
4	2008	M/49, respiratory	Sputum	Unknown	4	1	0.25	0.06	0.007	CLSI M38-A2	ND
5	2009	M/35, hematology	Sputum	Unknown	0.5	4	0.12	0.06	0.007	CLSI M38-A2	ND
6	2015	M/55, unknown	Sputum	Unknown	>16	1	>16	0.25	0.06	EUCAST	WT
					>16	2	1	0.5	0.06		WT
					2	2	1	0.5	0.06		ND
7	2016	M/73, necrotizing aspergillosis of the lung	Sputum	Yes	16	>16	4	0.5	0.03	EUCAST	TR_34_/L98H
8	2016	F/68, asthma, and bronchiectasis	Sputum	Yes	>16	2	0.5	0.5	0.06	EUCAST	TR_34_/L98H
9	2017	M/39, trauma, and Intensive care	BAL	No	4	0.5	0.12	0.25	0.03	EUCAST	TR_34_/L98H
10	2017	M/66, HIV, aspergilloma, and hemoptysis	Sputum	Yes	8	0.5	1	0.5	0.06	EUCAST	ND

*AFST, antifungal susceptibility testing; AMB, amphotericin B; CAS, caspofungin; CLSI, Clinical Laboratory Standards Institute; EUCAST, European Committee on Antimicrobial Susceptibility Testing; F, female; HIV, human immunodeficiency virus; ITC, itraconazole; M, male; MEC, minimum effective concentration; MIC, minimum inhibitory concentration; ND, not done; PCZ, posaconazole, VRC, voriconazole; WT, wild-type.*

All azole-resistant *A. fumigatus* isolates were cultured from sputum samples. Two patients had hematological underlying diseases, two had chronic respiratory disease and one had human immunodeficiency virus (HIV) infection. With the exception of one case (case 1) with unknown outcome, four cases died (case 2–5). However, we were not able to determine if the death was attributed to azole-resistant aspergillosis or the underlying clinical conditions in these patients. One azole-resistant isolate displayed a concomitant raised MEC (8 mg/L) to caspofungin. However, all azole-resistant isolates remained susceptible to amphotericin B. No clinical information on prior azole therapy was available on any of five patients. The first azole-resistant *A. fumigatus* from these five patients was isolated in 2001.

### Prospective Surveillance

From 2015 to 2017, a total number of 356 clinical isolates were identified as *A. fumigatus* over a 3-year period. Antifungal susceptibility testing (AFST) was conducted on 318 out of 356 (89.3%) isolates. Among isolates with AFST data, the majority (289/318, 90.9%) were cultured from either sputum (*n* = 179) or bronchoalveolar lavage (BAL) fluid (*n* = 110) samples.

Seven isolates showed MIC of ≥2 mg/L to itraconazole (2.2%). This ranged from one isolate being intermediate (MIC = 2 mg/L), two isolates had MIC of 4 and 8 mg/L and four isolates displayed high level itraconazole resistance (MIC ≥ 16 mg/L) (**Table 1**). Only one isolate demonstrated high level resistance to voriconazole (MIC > 16 mg/L) while the remaining isolates showed intermediate (*n* = 3) or susceptible phenotype to voriconazole (*n* = 3). While six isolates displayed resistance to posaconazole (MIC > 0.25 mg/L), high level of resistance against posaconazole was only detected in one isolate. Notably, one isolate from case 7, displayed a pan-azole resistant phenotype.

The seven azole-resistant isolates were recovered from five patients. All of the resistant strains remained sensitive to amphotericin B and caspofungin. All triazole-resistant isolates were cultured from respiratory samples. All clinical isolates with azole-resistant phenotype were confirmed as *A. fumigatus* using matrix-assisted laser desorption ionization–time of flight mass spectrometry (MALDI–TOF MS).

### Azole Resistance Prevalence

Overall, reduced susceptibility to triazoles antifungal agents remained low in a large collection of unselected clinical *A. fumigatus* isolates tested in our centralized mycology laboratory in London. In total, only 0.81% (12/1469) isolates with available AFST data displayed reduced susceptibility to at least one triazole antifungal agent over a period of 17 years. However, prevalence of azole-resistant *A. fumigatus* was increased from 0.43% in 1998–2011 to 2.2% in 2015–2017 (*P* < 0.05, Fisher Exact test). Within the study period, pan-azole resistance has been recorded amongst tested isolates, however its occurrence remains rare (*n* = 1).

### Mechanism of Resistance

From 12 clinical *A. fumigatus* isolates with azole-resistant phenotype, five isolates collected from 2015 to 2017 were available to investigate their molecular mechanism of resistance. When tested for mechanism of resistance using AsperGenius^®^ high resolution melt-curve analysis, TR_34_/L98H was present in three isolates (60%). Two isolates with azole-resistant phenotype did not show any alteration in TR_34_/L98H when tested by AsperGenius^®^ assay.

## Discussion

Although the prevalence of azole resistance in *A. fumigatus* has been investigated in diverse populations and in different countries (Hagiwara et al., 2016; Verweij et al., 2016b; Chowdhary et al., 2017), the true frequency of resistance in the United Kingdom remains largely unknown. This is predominantly because previous reports from United Kingdom were all based on studies (Howard et al., 2006, 2009; Bueid et al., 2010; Denning et al., 2011) carried out in the National Aspergillosis Centre in Manchester which represented a very specific patient sub-population predominantly with chronic forms of aspergillosis. In essence therefore this population did not represent other general and mixed patient cohorts in other centers with different underlying diseases such as hemato-oncology or solid organ transplantation. Furthermore there is no national surveillance program to actively screen clinical or environmental isolates for resistance to triazole antifungal agents in the United Kingdom. The prevalence of azole-resistant *A. fumigatus* may differ considerably from center to center depending on the geographical location of studied hospitals. To compound this, most clinical microbiology laboratories in United Kingdom either do not routinely test *Aspergillus* isolates for antifungal susceptibility testing or refer those isolates deemed clinically significant to reference laboratories. Expert recommendation by the recently instituted International Society for Human and Animal Mycology (ISHAM) *Aspergillus* Resistance Surveillance Working Group has highlighted the importance of performing local surveillance in order to determine the presence of azole resistance and adjust treatment guidelines if necessary (Resendiz Sharpe et al., 2018).

In the present study the prevalence of azole-resistant *A. fumigatus* recovered from clinical samples collected from unselected patient populations remains low. Among all clinical isolates tested from 1998 to 2011, only 0.43% (5/1151) demonstrated reduced susceptibility to at least one triazole antifungal agent. This figure increased to 2.2% (7/318) between 2015 and 2017, when a prospective passive surveillance program was carried out over a 3-year period in the same laboratory. This increase in the incidence of triazole resistance among mixed patient population, was in agreement with the general increase described in the recently published ESPAUR report (Public Health England, 2017). Arguably, the 8.5% itraconazole resistance among *A. fumigatus* isolates tested at the national reference laboratory may represent a bias due to testing clinical isolates that were predominantly originated from patients with refractory disease or in whom more exposure to medical triazoles was expected. Internationally, resistance prevalence in populations comprising of mixed patient groups is consistent with published data from countries such as Denmark (Jensen et al., 2016), France (Alanio et al., 2011), India (Chowdhary et al., 2015), Iran (Mohammadi et al., 2016), Pakistan (Perveen et al., 2016), Kuwait (Ahmad et al., 2014), Australia (Kidd et al., 2015), and United States (Berkow et al., 2018) where, overall, prevalence of antifungal resistant *A. fumigatus* remained low.

This study showed a clear difference in the prevalence of azole-resistant *A. fumigatus* in London when compared to the previously published data from NAC in Manchester (Howard et al., 2009; Bueid et al., 2010; Denning et al., 2011). This significant variation in the proportion of resistance, suggested that patients with chronic airway diseases might be at higher risk of colonization and/or infection with azole-resistant *A. fumigatus* when compared to general or mixed patient cohorts. Similarly, prevalence of azole resistance in clinical *A. fumigatus* in French patient populations was dependent on underlying clinical conditions; 1.1% in hematological patients, 1.8% in unselected patients (Alanio et al., 2011), and 8% to 12.2% in patients with cystic fibrosis (Morio et al., 2012; Guegan et al., 2018). This finding supports the recommendations by van der Linden et al. (2016) about the need to determine azole resistance frequency at the hospital level and within different patient groups. Additionally, recording clinical data to include triazole duration and dose administered for prophylaxis and/or treatment in conjunction with therapeutic drug monitoring will elucidate the potential relationship between previous azole exposure and development of antifungal resistance.

To investigate the common *cyp51A*-dependent mechanisms of resistance, DNA extracts from five available fungal isolates with azole-resistant phenotype were tested with AsperGenius^®^ multiplex RT-PCR assay followed with high resolution melt-curve analysis. TR_34_/L98H detected in 3/5 (60%). This is the first time that presence of this polymorphism has been shown in London. Two *A. fumigatus* with azole-resistant phenotype (both from case 6 isolated in 2015) did not demonstrate any TR_34_/L98H or TR_46_/Y121F/T289A alterations. Unfortunately none of the five azole-resistant isolates found between 2001 and 2009 were available for further analysis so we cannot, at this stage, determine whether the incidence of these alleles has increased across the period of study. Furthermore, recovering azole-resistant *A. fumigatus* from patients with retroviral and hematological underlying conditions in this study indicates that isolation of azole-resistance *A. fumigatus* is not limited patients with respiratory disorders.

Limitations of our study include the absence of data for a period of 3 years (2012–2015) when antifungal susceptibility testing was excluded from the routine diagnostic service. Molecular basis of azole-resistant in two isolates with no alteration in *cyp51A* gene remains unknown. Mutations in hot spots in *cyp51A* gene or other non-*cyp51A*-related mechanisms like efflux pumps might be responsible for elevated MIC values to triazoles in these two isolates. Furthermore, six azole-resistant *A. fumigatus* isolates were not available for molecular testing.

The current study identifies an overall low proportion of azole resistance (0.81%) in clinical *A. fumigatus* isolates obtained from a mixed and diverse patient population in London, United Kingdom. However, there are signs that this may be changing as there has been an increase in recent years showing that further cross-sectional studies are necessary to establish whether this trend is mirroring that seen in other European countries. It is also necessary to conduct similar surveillance studies in specific patient populations such as those with chronic respiratory diseases at regional level to investigate whether the prevalence of triazole resistance varies between different patient cohorts. The discovery of environmentally driven TR_34_/L98H among azole-resistant *A. fumigatus* isolates is of clinical significance suggesting a spillover of environmentally acquired antifungal resistance into susceptible patients. Systematic, continual and multi-center surveillance programs at a nation-wide scale are warranted to provide comprehensive epidemiological data on triazole-resistant *A. fumigatus* in United Kingdom.

## Author Contributions

AA, MCF, and DA-J contributed to the conception and design of the research. AA performed all experiments and wrote the manuscript. MAP carried out AFST on retrospective fungal isolates. All authors contributed to the analysis and interpretation of data and the drafting and revising of this manuscript.

## Conflict of Interest Statement

AA has been paid for talks and received travel support from Gilead. The remaining authors declare that the research was conducted in the absence of any commercial or financial relationships that could be construed as a potential conflict of interest.
